# Type 1 Diabetes in care homes: A practical guide on management

**DOI:** 10.1111/dme.15457

**Published:** 2024-11-05

**Authors:** A. J. Sinclair, S. Bellary, A. Middleton, A. Morris, R. Walker, K. Winkley Bryant, U. Dashora, M. Karamat, J. Rayner, S. Tomlinson, G. Maltese

**Affiliations:** ^1^ Foundation for Diabetes Research in Older People and King's College London UK; ^2^ University of Aston and University Hospitals Birmingham NHS Foundation Trust Birmingham UK; ^3^ Person Living with Diabetes (PLWD) and Diabetes, Diabetes Research Steering Group London UK; ^4^ Diabetes UK London UK; ^5^ Successful Diabetes Northampton UK; ^6^ King's College London UK; ^7^ East Sussex Healthcare NHS Trust and Joint British Diabetes Societies‐IP Care JBDS‐IP and Association of British Clinical Diabetologists (ABCD) Saint Leonards on Sea UK; ^8^ University Hospitals Birmingham Foundation Trust Birmingham UK; ^9^ Hallmark Care Homes Essex UK; ^10^ Medwyn Surgery Dorking and University of Roehampton London UK; ^11^ Epsom and St Helier University Hospitals NHS Trust, and King's College London UK

**Keywords:** age, care delivery, quality, type 1 diabetes

## Abstract

The primary purpose of the original NAPCHD project was to develop a national Strategic Document of Diabetes Care for Care Homes which has now been completed and well received as a worthwhile, sustainable, and effective guidance for delivering quality diabetes care in the UK. A Working Group of NAPCHD was established to produce a Position Statement on type 1 diabetes in care homes since this area was recommended as a topic to further develop. There are currently limited data on the prevalence and clinical outcomes associated with type 1 diabetes in care homes and management policies have been non‐existent in the UK. Communication among all key stakeholders involved in direct care of residents with type 1 diabetes is generally fragmented and lacks coordination. This is compounded by a slowly growing utilisation of diabetes technology and the absence of a standard/agreed community‐based model of interdisciplinary collaboration. The Rationale and Objectives were defined prior to commencing the work and a work plan with individual tasks was initially set out. After multiple correspondences and Team calls over a period of 9 months, the Group successfully generated a first draft in October 2023. This draft was then finalised the following month and circulated among stakeholders for feedback. Nine chapters have been provided including minimum standards of diabetes care, insulin regimens, avoiding hospitalisation and discharge planning. A scheme for a community‐based model of care for type 1 diabetes has been included. Eight key messages were developed. In addition, an Appendix has been created which includes key assessments such as nutritional assessment, detection of frailty, sick day rules and foot risk stratification (available online).

What's new

- This Position Statement is the first England‐wide multidisciplinary collaboration among representatives of some key diabetes societies, people living with diabetes (PLWD), senior nursing and medical consultants in diabetes, and stakeholders. Its aim is to offer guidance on managing type 1 diabetes in care homes in the UK.
- We provide the first comprehensive guidance on the implementation of insulin regimens that are both effective and safe within care homes.
- We have developed the first published set of glucose and HbA1c targets, and monitoring frequency for residents with type 1 diabetes for optimum safety, to prevent hyperglycaemia, minimise the risk of hypoglycaemia, infections and other acute complications.
- We present a community scheme for safe and effective transfer of residents with type 1 diabetes in and out of hospital.
- We provide a template on the essential elements of Education and Training of Care Staff on type 1 diabetes in Care Homes.

## 1 INTRODUCTION TO THE WORK OF THE WRITING GROUP

For over two decades, it has been well established that diabetes is a highly prevalent metabolic disorder in British care homes, affecting more than one in four residents^1^ The vast majority of these individuals have type 2 diabetes (T2D).^2^ Work of the National Advisory Panel for Care Home Diabetes (NAPCHD) indicated that residents with type 1 diabetes (T1D) have similar levels of vulnerability with high levels of frailty and multiple long‐term conditions as their counterparts with T2D.^3^ Like individuals with T2D, their illness, is often multifaceted, requiring varying levels of medical, nursing and social care needs.

Previous clinical guidelines and diabetes care policies relevant to care home residents with diabetes have not discussed management of T1D residents in any detail,^4^, ^5^, ^6^, ^7^, ^8^ although several local guidelines exist such as in the Wirral, UK: see https://www.wuth.nhs.uk/media/8835/diabetes‐policy‐with‐nice‐approval‐wirral‐care‐homes‐70618‐locked.pdf.

A number of shortfalls in care for T1D were identified during the original NAPCHD phase of this work.^3^ These included the absence of known or available standards of diabetes care, highly variable insulin regimens lacking optimal strategies, a lack of recommendations regarding monitoring frequency or the use of continuous glucose monitoring, and a notable absence of educational guidance for training care staff in managing type 1 diabetes. In particular, little guidance was available for managing end‐of‐life scenarios. In this respect, it is vital to have a clear view of how to balance quality of life issues and diabetes control, and sometimes it may be necessary for some residents in this scenario to be allowed some dietary indiscretions.

What is required are enhanced data gathering and auditing processes specifically focused on T1D in care homes. To date, there are no published data on T1D in care homes in the UK to our knowledge. Additionally, there is a need for improved communication and collaboration among all stakeholders involved in the direct care of the residents with diabetes, as well as greater use of technology which has many potential advantages for enhancing diabetes care within care home settings.^9^

These latter statements provided the *Rationale* for this Position Statement. We hope that many if not all of our recommendations for T1D will shed light on ongoing concerns within this sector and that once funded, actioned and deployed, will bring about positive changes that improve the lives of all care home residents with T1D living in the UK.

## 2 RATIONALE FOR THIS WORK

The rationale for this work can be summarised through a series of bullet points which enabled the Writing Group to focus their work on specific issues:

- There is little published work on T1D in community‐dwelling older adults and even less on care home residents with T1D diabetes.
- There is insufficient description of the key features of T1D in care homes.
- There is a need for developing *Minimum Standards of Diabetes Care* for managing T1D in care homes.
- There is a need to develop effective but safe and simple insulin regimens within care homes.
- There is an important need to agree glucose targets, HbA1c levels and monitoring frequency in residents with T1D for optimum safety, to reduce infections and other acute complications, reduce hyperglycaemia, minimise hypoglycaemia and unwanted hospital admissions.
- There is a necessity to establish a community scheme aimed at ensuring the safe and effective transfer of residents with T1D to and from hospital.
- There is a need to agree the essential elements of Education and Training of Care Staff on T1D in Care Homes.

## 3 MAIN OBJECTIVES

The Writing Group have identified four main objectives for their work which are summarised below:

- To raise awareness of the special features of T1D in care homes among care staff and community and primary healthcare providers.
- To develop a set of *Minimum Standards of Diabetes Care* for T1D in care homes
- To develop a set of *Key Management Strategies* for T1D in care homes.
- To propose a community‐based model of T1D care in care homes with special links to primary care and hospital services.

### 3.1 How did we go about this work?

Method—we convened a panel of experts initially comprising members from the original NAPCHD panel which included a care homes representative, a person living with diabetes (PLWD), a senior diabetologist, a representative of Diabetes UK, and a representative of the Association of British Clinical Diabetologists (ABCD). This panel was extended to include a senior academic nurse and a community diabetologist, both with a strong history of interest in diabetes in older people, and a further PLWD.

The Writing Group established nine task areas which included defining Minimum Standards of Diabetes Care for T1D in Care Homes. These individual task areas included those members who had suitable roles and responsibilities in professional practice, education and research currently.

The following 4 starting questions for the majority of task areas formed the basis of the work:

- What do we know about the current status of this issue—is current practice in this area of a high standard, is it organised, systematic, observable (current state)?
- What are the deficiencies of care, or needs or knowledge in this area?
- What key steps are needed to bring about worthwhile change—new knowledge, audit, training/education of care staff, enhanced networking, improved technology, behavioural change, funding, etc.?
- How can the key steps be realistically implemented?

### 3.2 Who should read this document?

It was our intention to produce this Position Statement on T1D for care home managers and care staff, primary care professionals (GPs, paramedics, pharmacists, advanced nurse practitioners), community diabetes specialist services, hospital inpatient, outpatient and emergency service staff, social care personnel, voluntary organisations engaged with care homes, independent care homeowners, local commissioning groups, and all members of the NAPCHD stakeholders involved in developing the main guidance which is available at: NAPCHD￼ – fDROP.

## 4 METHODS

Following the acceptance of invitations to join the Writing Group which were sent by the Chair, a briefing paper was prepared. Based on the feedback and discussion, the writing group identified key issues in type 1 diabetes management in care homes and identified defined objectives. Initially, every member was involved in developing minimum standards of diabetes care within a care home setting, and then four subgroups examined insulin regimens and glycaemic targets, evidence for continuous glucose monitoring (CGM) and monitoring, management of comorbidity and frailty, local implementation of the type 1 care pathway. All members contributed to the other chapters. Communication primarily occurred online, including at least three meetings of the type 1 panel organised by Orange Juice Communications, Northamptonshire. Additionally, individual meetings with the Chair were arranged. A first draft of the Position Statement was distributed to the Writing Group in late October 2023 and after further refinement it was finalised in November 2023 ready for circulation and comment from interested stakeholders.

This Position Statement by the NAPCHD on type 1 diabetes in care homes brings eight key messages:

- An urgent need to undertake more audit and clinical research including randomised controlled clinical trials (RCTs) in care home settings of residents with type 1 diabetes; this necessitates investment from national funding bodies and other sources to support research initiative focused on care homes.
- Consideration of data on residents with both T1D and T2D being included in a national audit of diabetes in care homes.
- A justified need to enhance education and training of care staff and involved health care and social care professionals in the basic requirements of caring for residents with T1D.
- Proposed minimum standards of care for residents with T1D across England are now available in this Position Statement.
- We have proposed an example of an integrated community‐based model of diabetes care for residents for the resident with T1D
- Provisional guidance on the CGM targets for residents with T1D is available in this Position Statement.
- A coordinated and integrated plan of diabetes care for residents with T1D discharged from hospital to a care home is available in this Position Statement.
- This Position Statement has an online *Appendix* which provides useful and user‐friendly assessment tests for care home staff in the areas of nutritional status, frailty, cognitive assessment, sick day rules, foot risk stratification and quality of life.

## 5 KEY RECOMMENDATIONS

For Type 1 diabetes, Commissioners of services should ensure:

- Access to specialist services is available to everyone with T1D throughout their lifetime, as needed. This should encompass a personalised diabetes review schedule, based on individual needs, including blood tests as necessary, and appropriate health assessments.
- This should include a diabetes review, with a frequency based on individual's needs, blood tests when warranted, regular blood tests and appropriate health assessments. At all stages, it is important to retain a pro‐active approach to anticipate clinical adverse events and changes in the health status of each resident with diabetes.
- Local arrangements for a structured programme aimed at promptly initiating education and insulin therapy upon diagnosis, as well as managing insulin or insulin pump therapy and training healthcare professionals and patients (this will include having access to the CGM NICE Guidelines, available at: Quality statement 2: Continuous glucose monitoring|Type 1 diabetes in adults|Quality standards|NICE).

These standards and quality guidance rarely apply to older adults with T1D and even rarely to those residents with T1D. One of the key purposes of this document is to raise awareness of the lack of equity in this area. In this Position Statement, we focus on minimum standards of diabetes care, insulin regimens and recommended glycaemic targets for residents with T1D.

Other recommendations relating to key assessment procedures are given in Appendix 1 (e.g., clinical frailty scale, sick day rules, foot risk stratification, test for cognitive impairment, etc.—full guidance online). Nutritional requirements, testing for ketones, monitoring of glucose levels including CGM, insulin pumps, avoiding hospitalisation and discharge planning are provided online at: http://fdrop.net/wp‐content/uploads/2024/05/FINAL‐EDITED‐COPY‐after‐FEEDBACK‐Working‐Copy‐of‐Type‐1‐Position‐Statement‐March‐2024‐AutoRecovered.pdf.

### 5.1 Recommended minimum standards of care for residents with Type 1 Diabetes living in care homes

Minimum standards of care ensure a consistent framework for managing residents with diabetes and form the basis of quality care provision and audit work.

Standards are shown that indicate a degree of responsibility to ensure they are met. In this document, they apply to: The care home management and its staff (CH); that are relevant to the individual resident (R); and Standards of competency in diabetes care that are relevant to individual care staff (CH_Comp_).

In order for these standards to be effective, there remains an important need to develop better local and regional coordination and communication among all key stakeholders involved in providing diabetes specialist care (e.g., hospital specialist teams, community diabetes teams), and care homes. Otherwise, this creates a level of suboptimal care that was recently highlighted by the Covid‐19 pandemic: please see: Guidelines for the management of diabetes in care homes during the Covid‐19 pandemic—PMC (nih.gov); and: The impact of the COVID‐19 pandemic on diabetes services: planning for a global recovery—The Lancet Diabetes & Endocrinology.

Improvements can be achieved via the expansion and operations of the Integrated Care System (ICS) working in liaison with primary care networks and prompted by the implementation of the Enhanced Health in Care Homes (EHCH) initiative. Consideration should be given to developing district or regional teams to coordinate and enhance the quality of diabetes care delivered.^3^

All new developments in T1D care practices in care homes should attempt to meet the key requirements of the *NAPCHD Philosophical Framework* in order to provide a high standard for clinical care protocols, preventative strategies, audit projects and research participation.^3^ This approach paves the way for the development of service specifications aimed at delivering community‐based T1D care within care homes. Specifications should adhere to the NICE Quality Standard on Diabetes Care and be suitable for inclusion in contracts with local authorities (via Department of Health and Social Care, DHSC, available at: Overview|Type 1 diabetes in adults|Quality standards|NICE).

In Table 1, we have presented the list of standards and relevant information.

**TABLE 1:** NAPCHD minimum standards of diabetes care: Type 1 diabetes in care homes.

Standard	Comments
All residents with diabetes should be easily identified (CH)	A method for easy identification should be available and should indicate what type of diabetes the resident has (colour code or sticker to be used on/in care records). Contact details for community or hospital specialist support, where needed, should be easily accessible.
All residents with T1D in care homes should have an agreed personalised care plan (R/CH) *Part of this plan must include the contact details of specialist care providers (including deputies to cover absences) and the required frequency of specialist clinical reviews determined by primary care GP/diabetes specialist team plus the annual review date must also be included*.	Information contained within this care plan can be summarised within a ‘pen‐picture’ or on the care plan ‘front‐profile page’. Details needed in the files should include medication list, personalised targets for capillary glucose levels and HbA1c, individualised target blood pressure (taking into account frailty), hypoglycaemic awareness, risk of falls, frequency of capillary glucose monitoring (finger‐prick testing), CGM (continuous glucose monitoring)/flash glucose monitoring (FGM) readings, dates of last eye and foot screening should be included in this file. When appropriate pathways exist, this should also record their current *insulin‐to‐carbohydrate ratio* and *insulin sensitivity factor* for calculating dosages where staff are administering insulin (doses that are estimated according to a meal's carbohydrate content).
All residents should have an additional personalised nutrition care plan in their case files (R/CH) which is reviewed annually by a registered dietitian	This is important in residents who are frail or have borderline malnourishment (see MUST score in Appendix 1, online) and the maintenance of weight is essential.
All residents must be regularly checked for their self care skills: this will form part of their overall functional status assessment. (CH_Comp_)	This will include detecting frailty (consider using the CFS as it is pictorial) for the first time or level of frailty (if previously diagnosed) and cognitive status. Primary care GP, community diabetes nurse specialist (DSN) and annual review process can all help in capturing this information and then sharing and keeping this information on the patient record.
Each resident should carry a passport, for example, the one proposed by NAPCHD. (R/CH)	All care homes should have a champion who is familiar with CGM and flash glucose monitoring including aspects such as sensors replacement, and basic concepts of data interpretation. The champion can be a nurse or a non‐clinical carer and will need to be appropriately trained and skilled on a regular basis. Care home staff should be able to upload flash glucose data on to a device in each care home.
All residents should have an annual review in line with national guidance. (CH/CH)	Some residents may receive their annual review including checks for feet, eyes, review of medications, stopping medications that may be useful (a phase of de‐escalation) and annual review of blood tests in the care home. Advice from the primary care team or community diabetes specialist services may be required.
All homes who have residents with diabetes (both T1D and T2D) should have at least one member of the care staff with ‘with an interest in type 1 diabetes?’. (CH/CH_Comp_)	Ideally, this person will have a valid yearly (or two‐yearly if necessary) certificate of training and education in T1D [see links to the courses available in the main document]. This may be the care home diabetes champion or another individual.
All homes should have at least half a day per year of education and training in T1D for all their staff (and more where staff turnover is high). (CH)	This half day training could/should be delivered by a diabetes specialist service and requires negotiation and consideration of funding issues. Care homeowners should be responsible for ensuring staff are appropriately trained to meet the needs of residents, similarly any LA or NHS facilities would have the same accountability Where this is not possible/feasible an online module from a trusted source can be a next best alternative such as Diabetes UK (online modules including the Understanding Diabetes module) and Trend Diabetes.
All care home staff providing personal care should be aware of the importance of identifying and treating hypoglycaemia symptoms. (CH_Comp_)	Hypoglycaemia should be promptly treated.
All homes should have a ‘Hypo boxe’ that is within date and have fast acting glucose as per needs and preferences of their residents. (CH)	Hypo boxes should be inspected regularly (ideally weekly) for their content and expiry date of enclosed items. Access to Glucogel and glucagon is essential. Preferred hypo treatments should be always kept within easy reach of residents.
The ‘hypo awareness’ of people with T1D should be assessed during the planned diabetes review be known and understood by all staff. (CH_Comp_)	Hypo‐awareness of people with diabetes should be regularly reviewed using a simple method such as the Gold score asking a simple question whether the person knows when hypoglycaemia is starting on a linear scale (1 = always aware, 7 = never aware). Preferred hypo treatments should be always kept within easy reach of residents.
All care homes should ensure that care staff are able to detect early signs of hyperglycaemia and testing for urinary and blood ketones. (CH)	There should be available clear guidance on what steps to take in cases of worrying hyperglycaemia.
All care homes should ensure that people with T1D receive annual foot check and eye screening. (CH)	Feet should be inspected daily, and any sign of infection, skin discolouration or swelling should be brought to the attention of the local care foot pathway team, and treated urgently. Care records should have a section to record foot checks rather than just writing in the notes. For residents who are mobile, in order to maximise mobility, and reduce the risk of foot ulceration, falls/fractures and hospital admissions, it is imperative that footwear is supportive and well fitting. Any deterioration in vision should be reported to the senior staff of the care home. Whilst annual screening is advised as a minimum, testing as part of a routine screen can be arranged more frequently as required, for example, 6 monthly if there are active problems. Eye screening can sometimes be challenging for older people with advanced cognitive impairment, or physical difficulties that make it impractical. Something also about being fit enough (in the broadest sense) for onward referral for treatment if that might be advised, and whether that would be practically possible. Bear in mind that there is a range of care homes. A residential home may care for people who maintain good cognitive function and self‐caring ability, whereas there are other units where residents may be partially or completely dependent, or may have challenging issues due to advanced disease. Please see below new national guidance in retinal screening.
Psychology support (if available) should be provided by the psychologist within the community diabetes multidisciplinary team. (CH)	Residents who move to care homes may lose self care skills and independence after many years. The psychological impact of the loss of self care skills in T1D is huge and often greater than in T2D. Other forms of support should be accessed if available, for example, anxiety and depression support, e.g., NHS talking therapies.
All care homes should develop an audit tool to collect data on a regular basis. (CH)	This will include data on the frequency of hypoglycaemia, annual review of their residents, complications during the stay, self management capabilities of the residents among other audit parameters. An audit tool is available in the NAPCHD Appendices. Care homes should undertake the audit but be guided by the primary care team and community diabetes team by negotiation and funding considerations: Care Home Audits—What are the Key Principles to Follow (theaccessgroup.com) Safe management of the care environment compliance: Monthly Audit Tool for Care Homes—Infection Prevention Control Care Audit Tool—Auditing Software for CQC Compliance

### 5.2 Insulin regimens for care home residents with Type 1 Diabetes

We have provided a set of insulin regimens which we feel are suitable for residents of care homes with T1D (Table 2). Insulin therapy is always and strictly necessary in older people with T1D to avoid hyperglycaemia and diabetic ketoacidosis. Insulin treatment strategies should be individualised according to health status and presence and severity of frailty and life expectancy.

**TABLE 2:** Insulin Regimens for Care Home Residents with Type 1 Diabetes Mellitus.

Non frail/mildly frail resident	Moderately frail resident	Severely frail resident	Resident at end of life	Resident on enteral feeding	Resident on steroids (prednisolone or dexamethasone)
Insulin Detemir twice daily + rapid acting analogue (lispro/aspart) or continuous subcutaneous insulin infusion (CSII)	Once daily Degludec or Gla‐300 + rapid acting analogue if the person resides in a nursing home (facility) and the insulin is administered by care home staff	Once daily Degludec or Gla‐300 + rapid acting analogue (given PRN—when required) to achieve appropriate glucose levels target	Degludec or Gla‐300 given once daily	If the resident is on bolus tube feed the insulin regimen can include either Glargine or Degludec given once daily or Detemir given twice daily and rapid acting insulin with each bolus	If the resident is prescribed a course of prednisolone, NPH insulin can be added to the usual regimen in the morning at a dose based on weight and dose of prednisolone for the duration of the steroid treatment. If the resident is already on NPH or pre‐mixed insulin, the dose can be titrated according to glucose levels and targets
Degludec + rapid acting analogue	Degludec or Gla‐300 in the morning with rapid acting analogue at breakfast and dinner if insulin is administered by district (community) nurses			If the resident is on continuous nasogastric tube feed the insulin regimen can include Glargine or Degludec once daily or Detemir twice daily and rapid acting analogue every 4 h or regular insulin every 6 h	If dexamethasone is the steroid used, basal and/or rapid acting insulin should be titrated according to glucose levels and targets
Pre‐mixed insulin twice daily if it meets resident's needs and/or preferences	Pre‐mixed insulin twice daily if this regimen meets the resident's needs and/or preferences, or if insulin is given by district (community) nurses. Alternatively, NPH insulin twice daily ± rapid acting analogue				

*Note*: Baseline legend to Table 2: Please note the future changes in the availability of Levemir (detemir) which is mentioned in the text.

If the resident with T1D still has preserved self care skills, a basal bolus (or Multiple daily injections [MDI]) regimen with a long‐acting insulin twice daily (e.g., determir) or once daily (e.g., glargine 100 units/mL) and rapid‐acting analogue with meals (according to their individualised treatment plan) is often the gold standard approach to treatment. However, it should be noted that determir will be discontinued by 2026, so alternative long‐acting insulin will have to be considered.

A review of individualised treatment of a resident with type 1 diabetes often results in a need to simplify the insulin regime. An ultra‐long‐acting insulin (e.g., glargine 300 U/mL [Gla‐300]) with pre‐meal rapid‐acting insulin represents a valid alternative and is recommended for older people at risk of nocturnal hypoglycaemia or for frail individuals needing help from a carer or health professional to administer insulin.

Most adults with T1D use an insulin‐to‐carbohydrate ratio and a correction factor to calculate their meal‐time dose. This strategy, when applied to care home residents, may be compromised by the loss of numeracy associated with ageing and geriatric syndromes including cognitive impairment and care home residents with T1D may benefit from the assistance of a carer or registered nurse to estimate their meal‐time dose based on pre‐established meal carb content. An explanation of this ratio/correction factor is available at: 6403AdvancedInsulinManagementFinal.pdf (wcu.edu).

Insulin type and regimen changes should be undertaken in liaison with a diabetes specialist clinician (DSN or Diabetes Consultant) or by a healthcare provider with competencies in diabetes care including insulin management (Special interest GP, pharmacist, or dietitian), especially in relation to intercurrent medication (e.g., steroids above).

In Table 3, we present suggested glycaemic parameters for residents with type 1 diabetes based on health/functional status and presence of frailty. In the absence of clinical trial evidence, these represent expert recommendations only. The use of technology (e.g., continuous glucose monitoring (CGM)) to replace conventional ‘finger‐prick’ monitoring can provide significant advantages both to the carer and care staff but this will have to be proven in further studies. The use of insulin pumps and Hybrid Closed‐Loop systems in these settings will require much more evidence before implementation from well‐designed clinical trials. This is covered in more detailed in the online full guidance available at: http://fdrop.net/wp‐content/uploads/2024/05/FINAL‐EDITED‐COPY‐after‐FEEDBACK‐Working‐Copy‐of‐Type‐1‐Position‐Statement‐March‐2024‐AutoRecovered.pdf.

**TABLE 3:** Recommended glucose levels and HbA1c targets for care home residents with T1D according to health status and frailty.

Health status and frailty of the resident	HbA1c target (mmol/mol, %)	Fasting and pre‐meal glucose levels (mmol/L, mg/dl)	Bedtime glucose levels (mmol/L, mg/dl)
Healthy and no frailty Clinical Frailty Scale (CFS) 1–2 No major comorbidities No previous history of severe hypos	53–58 mmol/mol 7.0%–7.5%	6.0–8.0 mmol/L	6.0–9.0 mmol/L
Intermediate health status and moderate frailty CFS 6 ADL difficulties and 2–3 comorbidities; need some carer input No previous history of severe hypos	58–64 mmol/mol 7.5%–8.0%	6.0–9.0 mmol/L	6.0–10.0 mmol/L
Complex health status and severe frailty CFS 7–8 Dependent but may be relatively stable or in very severe cases, approaching end of life Reduced hypo‐awareness or previous severe/nocturnal hypos	68–75 mmol/mol 8.0%–9.0%	7.0–10.0 mmol/L	8.0–11.0 mmol/L
End of life CFS 9; life expectancy <6 months	Less than 15 mmol/L	Avoid hyperglycaemia	Avoid hyperglycaemia

### 5.3 Type 1 Diabetes care pathway in care homes: key features and place in an integrated model of community care

The responsibility for providing high‐quality care for residents with T1D must be shared among various stakeholders including care home managers, primary care providers such as GPs, community nursing teams and diabetes specialist teams operating in the hospital and the community.

**FIGURE 1:**
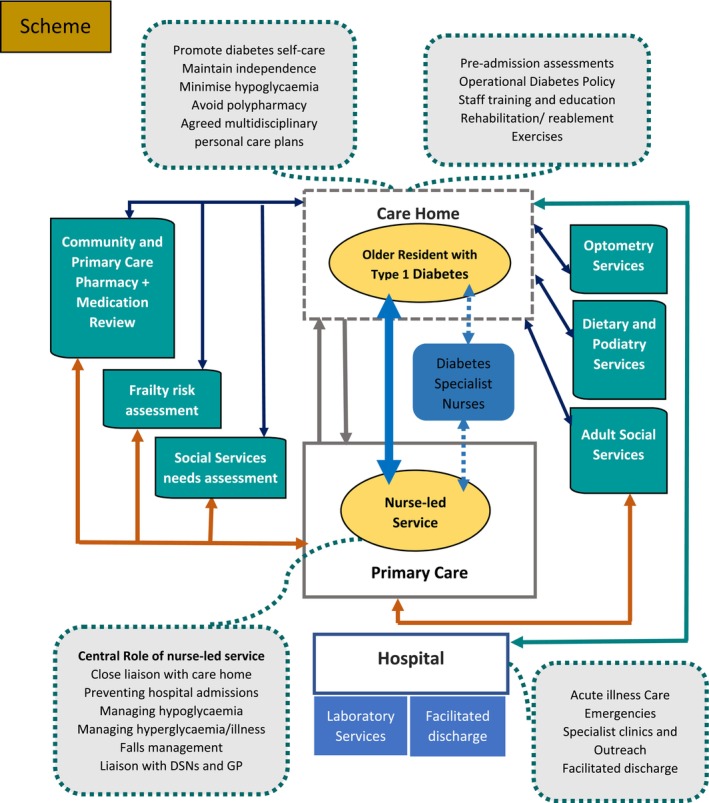
A community‐based integrated care model for care home residents with Type 1 diabetes. Adapted from the NAPCHD plan available at: http://Fdrop.net/wp‐content/uploads/2024/05/FINAL‐EDITED‐COPY‐after‐FEEDBACK‐Working‐Copy‐of‐Type‐1‐Position‐Statement‐March‐2024‐AutoRecovered.pdf.

The resident with diabetes, the care home, adult social services and the nurse‐led facilitator of diabetes care within the GP‐Primary Care Unit are at the heart of a model of care we propose (Figure 1) supported by all local key players such as community and specialist services, along with essential factors and other key stakeholders. It should be emphasised that no universal model exists and additional services such as dental care, nutritional support and skin integrity services support all have important roles in a care home setting. The role of Social Services and the NHS in jointly providing care needs to be established locally such as joint physio/OT assessments. In the cases of acute illness, it is important to emphasise the need for rapid assessment, and access to hospital and community‐based frailty clinics. The inter‐relations and communication channels are shown as interrupted lines. Support by a local primary care network (PCN) for senior primary care support and the community diabetes service should be encouraged. To avoid duplication in some areas, and to facilitate the best use of resources, a primary care diabetes nurse working within a PCN where available, may be able to support this model of service for care homes. Every effort should be made by practices to ensure that all residents with diabetes receive all the key care processes and interventions they would need in the QOF (Quality Outcomes Framework) model of primary care—see Quality and outcomes framework (QOF) (bma.org.uk) for further information.

## 6 FINAL COMMENTS

This work represents the first practical guidance on managing T1D in care home residents in England. Full guidance is available online. In the UK, our dissemination plan will involve (a) working through NHS partners, (b) direct communication with care homes, (c) engagement of diabetes charities, (d) through publication in journals, presentation at educational events and social media. We will share the guidance with the clinical networks for wider dissemination within the health service which will include the Care Quality Commission and Integrated Care Boards. Other European healthcare systems should plan a similar comprehensive dissemination plan. Finally, all registered care homes in England will be provided a copy of the guidance via the Care England Network and other means.

## AUTHOR CONTRIBUTIONS

A.J.S., G.M. and S.B. prepared the format of the manuscript; all authors contributed original content, and A.J.S. G.M. and S.B. produced first draft. All authors reviewed and had the opportunity to comment on the preparation of the final draft.

## CONFLICT OF INTEREST STATEMENT

No funding was received to prepare this work. No potential source of conflict of any sort was reported by any of the authors in the preparation of this work.
